# Smart Health-Enhanced Early Mobilisation in Intensive Care Units

**DOI:** 10.3390/s21165408

**Published:** 2021-08-10

**Authors:** Maria Ferre, Edgar Batista, Agusti Solanas, Antoni Martínez-Ballesté

**Affiliations:** Department of Computer Engineering and Mathematics, Universitat Rovira i Virgili, E43007 Tarragona, Catalonia, Spain; maria.ferre@urv.cat (M.F.); edgar.batista@urv.cat (E.B.); agusti.solanas@urv.cat (A.S.)

**Keywords:** early mobilisation, context-aware computing, sensors, smart health

## Abstract

Critically ill patients that stay in Intensive Care Units (ICU) for long periods suffer from Post-Intensive Care Syndrome or ICU Acquired Weakness, whose effects can decrease patients’ quality of life for years. To prevent such issues and aiming at shortening intensive care treatments, Early Mobilisation (EM) has been proposed as an encouraging technique: the literature includes numerous examples of the benefits of EM on the prevention of post-operative complications and adverse events. However, the appropriate application of EM programmes entails the use of scarce resources, both human and technical. Information and Communication Technologies can play a key role in reducing cost and improving the practice of EM. Although there is rich literature on EM practice and its potential benefits, there are some barriers that must be overcome, and technology, i.e., the use of sensors, robotics or information systems, can contribute to that end. This article reviews the literature and analyses on the use of technology in the area of EM, and moreover, it proposes a smart health-enhanced scenario.

## 1. Introduction

Post-Intensive Care Syndrome (PICS), or Intensive Care Unit Acquired Weakness (ICU-AW), is described as the disability that remains in patients surviving a critical illness after a long period in the ICU. Common symptoms include impairments in the physical function (e.g., fatigue, decreased mobility, etc.) to psychological health issues (e.g., anxiety, sleep disorders, cognitive issues, etc.). PICS, which is strongly associated with prolonged mechanical ventilation and bed-restore deep sedation, has been reported to occur on average in at least a quarter of ICU survivors [1].

**Figure 1:**
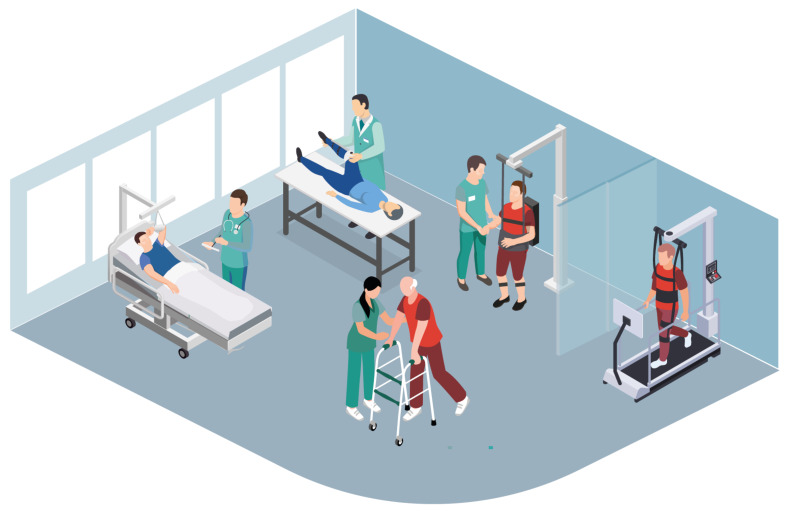
An Early Mobilisation scenario in a healthcare facility: patients in blue are undergoing passive mobilisations with the assistance of physical therapists (in green), and patients in red are performing active mobilisations.

Aiming at preventing PICS, Early Mobilisation (EM) protocols have been designed and are being applied during the patients’ stay in the ICU. Such protocols consist of routines of mobilisations and exercises: from passive mobilisation of limbs and postural changes for sedated patients to walking and reproducing basic everyday movements, including active mobilisation of limbs and even electrostimulation. Figure 1 depicts a general scenario in a healthcare facility where patients undergo EM protocols. Morris et al. [2] first proposed a four-level protocol for the progressive mobilisation according to the conscious or unconscious state of patients, their cardio respiratory stability and muscle strength in their limbs. Protocols typically begin with a safety screening: patients must meet all criteria in order to start the EM routines. First stages of protocols, for unconscious patients, are based on passive mobilisations and patient turns. Once patients are conscious, they are required to perform active resistance exercises and change to sitting position. Final stages of EM protocols include active transfers to chairs, ambulating in hallways and using ergometers.

A large body of literature is devoted to describing different approaches to EM, as well as to analyse their effects. A general consensus exists on EM being beneficial (i.e., patients have fewer ventilator-dependent days, shorter ICU and hospital stays, and better functional outcomes), and hence, it should be incorporated into daily clinical practice [3]. Last but not least, beyond generating substantial clinical improvements for ICU patients, EM reduces costs to hospitals [4]. However, the literature also considers several issues that require attention. Primarily, the wide variety of protocols and the different nature of ICU patients make it difficult for practitioners to adhere to a specific EM protocol. Furthermore, they prevent researchers from accurately studying EM effects and benefits. In this line, the optimisation of patients outcomes will require further studies on mobilisation timing and intensity, particularly within specific ICU populations [5]. Moreover, there are a number of elements that are perceived as barriers to the widespread implementation of EM, which are discussed next.

### 1.1. Barriers to Early Mobilisation

The literature on EM is essentially focused on the effects and potential benefits of such procedures: plenty of studies, reviews and surveys consider EM beneficial. Notwithstanding, a number of studies aim to identify the barriers to EM practice and its drawbacks. Aiming at identifying such issues, several studies use questionnaires [6,7] and identify three levels of barriers [8,9]:

- Patient-level barriers: related to patient safety and efficacy of EM (e.g., medical instability, endotrach intubation, obesity, cognitive impairment).
- Institutional-level barriers: such as a lack of equipment or unclear guidelines.
- Provider-level barriers: such as limited staff (issues typically reported by physical therapists), problems in communication and protocol continuity over shift changes.

Regarding the latter, experts suggest focusing on strategies such as increasing staffing and purchasing EM equipment in order to run successful EM programmes [9]. The sustainability of EM protocols has been described as essential for the successful implementation of EM, along with multidisciplinary team collaboration, data collection and feedback systems. Unfortunately, the intense daily activities in the ICU make fluid communication and appropriate data annotation puzzling. Nurses, physicians and physical therapists must proceed with EM protocols described on a document and must face monitoring with a scarcity of resources. All in all, communication between practitioners is essential to conduct safe and effective EM [10]. Moreover, the engagement of practitioners, patients and family is also considered a key component for EM practice, to which better communication could contribute [11].

### 1.2. The Evolution of Healthcare Paradigms

Information and Communication Technologies (ICT) have attracted the attention of the healthcare community: biometric sensors, robotics, electronic health records, etc., are concepts adopted in the everyday practice of medicine and healthcare. Electronic healthcare (or e-health), that is, “health services and information delivered or enhanced through the Internet and related technologies” [12], has become an essential cornerstone in medicine. E-health enables the provision of online medical treatments, the exchange of electronic health records in a standardised way between healthcare providers, and remote communications between patients and practitioners [13]. More recently, the advent of smartphones has resulted in their acceptance as important tools in the healthcare practice. In this line, mobile healthcare (or m-health), which could be defined as the use of “emerging mobile communications and network technologies for healthcare systems” [14], helps improve the communication between patients and practitioners, enables remote- and self-monitoring and defines medicine in a highly personalised, patient-centric perspective [15,16].

Along with the abiding implementation of ICT in the healthcare domain, cities started to equip their infrastructures with ICT to face both urbanisation challenges, such as the increase of population moving from rural areas to urban areas, and demographic challenges, such as the world’s population increase in life expectancy. ICT build up cities with sensing and analysis capabilities able to gather and exploit data in real-time to help decision-makers rearrange resources more efficiently and enhance the quality of life of citizens. Consequently, cities are steadily being transformed into smart cities, wherein citizens can interact with their immediate environment. By generalising this concept, environments capable of adapting themselves to users needs are commonly known as context-aware environments. Smart homes, smart buildings, smart hospitals and smart cities are examples of context-aware environments. The natural synergy between e-health and m-health converged with the emergence of smart cities and context-aware environments, and inspired smart healthcare (or s-health), introduced in [17] as “the provision of health services by using the context-aware network and sensing infrastructure of smart cities”. Although the provided definition was inspired within the scope of smart cities, the concept goes beyond their boundaries, and it could be generalised to context-aware environments. In addition to the patient-centric perspective used by both e-health and m-health paradigms, s-health also introduces an environmental/contextual approach, as it considers data coming from the sensing infrastructure of the context-aware environment and adapts its behaviour accordingly [18,19]. The generalised use of the Internet of Things (IoT) and low footprint sensors, which are able to gather vast amounts of contextual data with reduced computing capability and energy consumption, can contribute to the massive deployment of smart, context-aware healthcare solutions at large [20].

### 1.3. Contribution and Plan of the Article

This article discusses how technology, i.e., the use of sensors, information systems, biomedical electronic devices, etc., can contribute to the improvement of the practice of EM. Specifically, we aim to answer the following research questions:

- Q1: Does the relevant literature on EM practice consider the use of technology?
- Q2: Does the literature on ICT include applications to EM?
- Q3: How can technology (e.g., e-health, sensors, robotics, etc.) help overcome barriers to EM practice?

To answer these questions, we have adopted the methodology proposed by Vom Brocke et al. for conducting literature reviews [21]. According to this methodology, we have addressed the definition of the review scope, conceptualised the topic and conducted the literature search. Section 2 describes these first phases, specially the identification of the relevant literature. Answering Q1 and Q2, in Section 3, we have analysed the selected literature by highlighting the aspects related to technology. Next, Section 4 answers Q3 through presenting a scenario for enhancing EM via technology and the smart healthcare paradigm. Finally, Section 5 provides some conclusions and final remarks.

## 2. Methodology

In this section, we describe the process of selecting the literature that will be analysed to address Q1 and Q2. Next, we define the review scope, discuss on the conceptualisation and describe the selection methodology.

### 2.1. Definition of the Review Scope

The focus of the review is to identify articles that are focused on the relationship between technology and EM, e.g., by considering technology as an important factor in EM practice or by presenting technological solutions aiming at improving EM. Our goal is to select and synthesise the literature on the topic so as to depict the use of technology in EM globally. We will organise results by grouping proposals and adopting a neutral but critical position. The review is intended for both practitioners in the healthcare area and professionals in the sector of ICT, which aim to comprehend how technology can contribute to EM success.

### 2.2. Conceptualisation

According to [22], ‘early mobilisation’ is the most frequently used term for the topic. However, this article clarifies that ‘early rehabilitation’ is often used in some articles, especially in Europe. Some other infrequent terms in the literature are ‘early activity’ or ‘early physical therapy’, among others. Furthermore, there is some literature that addresses the early mobilisation of extremities after injuries or surgeries. For the sake of clarity, we have concentrated on the concept of ‘early mobilisation’ or ‘early rehabilitation’ whenever they refer to protocols aiming at performing *“a range of bodily movements carried out by care provider as part of care for a critically ill patients admitted into the ICU, which may include active or passive movement in bed, sitting up in bed, sitting by edge of bed, early transfer out of bed, sitting and standing out of bed, ambulation, …”*, as described in [22]. Hence, articles addressing issues such as rehabilitation after injuries will be considered out of the scope.

Technology is a broad concept that encompasses a number of terms and disciplines: from simple mechanical equipment to the widely-spread ICT, namely computerised information systems, mobile applications, robots or sensors, to name a few. Since one of the identified barriers to EM is the appropriate monitoring and information acquisition on the practice of EM routines, the so-called Human Activity Recognition discipline (HAR) can play a key role. HAR is the automatised detection and understanding of actions performed by individuals [23], which can be applied in a number of different areas, e.g., ambient assisted living, surveillance, physical rehabilitation, etc. Its development is supported by software in the fields of computer science and machine learning and by sensors (cameras, accelerometers, etc.) as well. Another technology-related discipline that may contribute to EM practice is robotics. For instance, robots can help perform passive mobilisations of extremities. Furthermore, software can contribute to overcoming many of the barriers to EM practice, e.g., from applications aiming to ease the adherence to EM protocols to applying data mining or process mining techniques. These aspects will be further addressed in Section 3.

### 2.3. Literature Selection

Our approach to literature selection is aimed at obtaining three sets of contributions:

- Set 1. The most recent reviews and surveys on EM that consider/mention technology in their analyses or discussions. The articles in this set will be used to address Q1.
- Set 2. The most-cited contributions on EM that mention the use of technology. The articles in this set will also be used to address Q1.
- Set 3. Original research articles on the application of technology to EM. The articles in this set will be used to address Q2.

**Figure 2:**
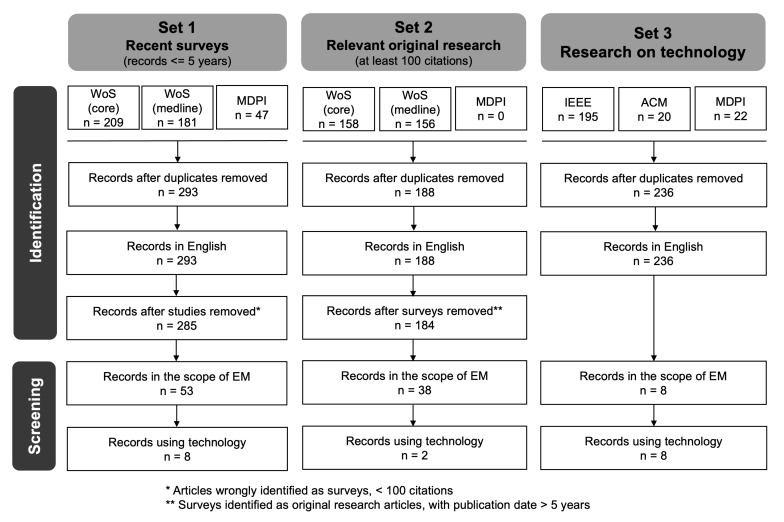
Synthesis of the search, screening and selection methodology.

To this end, a two-phase review methodology, consisting of an initial search phase and a latter screening phase, has been followed. This methodology is depicted in Figure 2. First, the search phase aims to identify a preliminary list of potentially relevant works and, to this end, some queries were designed and executed on scientific literature databases. Regarding Set 1, we searched the Web of Science (WoS) databases, specifically WoS—core collection and WoS—medline (the primer life sciences database), using the following search string:

S1 = (topic = {“early mobili*ation” OR “early rehabilitation”}) AND (title={“review” OR “survey”}).

The *topic* field includes title, abstract, author keywords and *keywords plus* (https://support.clarivate.com/ScientificandAcademicResearch/s/article/KeyWords-Plus-generation-creation-and-changes, accessed on 1 May 2021). Wildcard * is used to query using both spellings of the same word (e.g., mobilisation, mobilization). Due to the amount of results (771 results) and aiming at focusing on recent surveys, we selected articles published in the last 5 years.

Regarding Set 2, we used the following search string:

S2 = (topic = {“early mobili*ation” OR “early rehabilitation”})

Note that the number of citations can be considered as a measure of the quality of a publication [24]. In order to select the most cited-works, we only considered articles with more than 100 citations according to WoS.

To obtain the preliminary candidates for Set 3, which focuses on technology applied to EM, we conducted searches in IEEE Xplore (which indexes documents from publications in ICT areas, e.g., computer science, telecommunications or electronics) and the ACM Digital Library (that provides access to documents in computing-related areas). We have used the following search string:

S3 = (fulltext&metadata = {(“early mobili*ation” OR “early rehabilitation”) AND (“HAR” OR “human activity recognition” OR “sensors” OR “robotics” OR “software” OR “artificial intelligence”)})

For Set 2 and 3, we did not apply any timespan criteria to our search. For Set 3, the search was done over the entire text and metadata. Searches were conducted on 31 May 2021. Duplicated or non-English articles were removed. Moreover, some of the articles were misidentified: eight original research articles were obtained for Set 1 and were not included into Set 2 because they had less than 100 citations; similarly, four surveys were removed from the results of Set 2 and could not be included in Set 1 because of their publication date.

In addition to these searches, we conducted a selection of articles using the MDPI database. Specifically, the following search string was used:

$S_{add}$ = (allfields = {“early mobili*ation” OR “early rehabilitation”})

This search produced 238 results. In the identification phase, title and abstracts were revised in order to classify the articles as recent surveys, original research articles or articles on technology.

After the identification phase, two of the authors performed a blind screening, whereas other authors contributed to breaking ties: hence, if the two reviewers have different opinions, a third one participates in the final decision. For Set 1, full articles were screened (whenever the full paper was available) to assess to what extent they consider technology, i.e., the survey does not mention the use of technology, the survey highlights some uses of technology, or the survey considers technology in the analysis and classification of studies. For Set 2, titles and abstracts were reviewed to determine whether the articles fit with our concept of EM: for instance, articles addressing the mobilisation of joints after injury, articles addressing post-operative procedures and articles related to newborn ICUs were identified as out of the scope. Moreover, the screening phase filtered out articles not addressing technology. Finally, for Set 3, both titles and abstracts were screened. However, for Sets 2 and 3, the full-text of the paper was reviewed, if available, in case titles and abstracts did not provide enough information to classify the paper.

## 3. Results

After the screening phase, Sets 1, 2 and 3 contained eight, two and eight articles, respectively, as shown in Table 1. In this section, we overview the main findings after analysing these articles.

### 3.1. Technology and Early Mobilisation

One of the most cited works on EM addressing technology is [26], which has 127 citations. The article explores relevant technologies for rehabilitation of critically ill patients to prevent neuromuscular complications, namely neuromuscular electrical stimulation (NES), cycle ergometry and custom-designed technological aids and equipment for ambulation. The first two methods are more convenient at the early stages of the rehabilitation (even for sedated patients), whereas the latter requires the active participation of patients. As the latter is more challenging, authors presented a holistic system that improves the safety and effectiveness of ambulation of mechanically ventilated patients in the ICU, while reducing the number of human resources required to perform this task. Note that this article presents the outstanding technology that will be predominant in EM settings: on the one hand, passive EM using neuromuscular electrical stimulation, which creates passive contraction of muscles through the use of a low-voltage electricalised impulse delivered through electrodes; and on the other, active EM using cycle ergometers, i.e., stationary cycling apparatus with built-in mechanisms that can alter the exercise done by patients. Note that both cycle ergometers and electro-stimulators can to be programmed to perform a set of routines, with diverse intensities and lengths and, hence, can help practitioners apply precise and personalised EM protocols.

Physical therapists play a key role in the practice of EM. In order to asses the use of technology and equipment, ref. [25] presents the results of a survey completed by 206 practitioners. A total of 74% of respondents reported using at least one type of equipment or technology. Meanwhile, 31% of respondents reported using cycle ergometers, whereas the most common reason for not using equipment or technology was their limited or no availability. Approximately 75% of physical therapists emphasised the need for further research to better understand clinical approaches to the use and efficacy of technology.

#### 3.1.1. Neuromuscular Electrical Stimulation

A number of articles consider NES as a pivotal element in EM protocols. In [34], which has 144 citations, authors concentrate on the use of NES in a trial where 196 patients underwent an EM intervention. Nevertheless, the approach of this article is slightly different since it concentrates on the outcomes instead of discussing the additional support of technology to EM. However, it is worth noticing that patients could continue with post-discharge training using electric stimulation at home. The surveys in [30,33] relate 11 cases in which the effectiveness of NES is evaluated. In general, this technique is applied in 30 min sessions once or twice a day. Last but not least, NES is also described as a successful and interesting practice in EM for patients with COVID-19 [27,28].

#### 3.1.2. Robotics and Mechatronics

A robotic tilt table that allows early mobilisation of bed-ridden patients through modulating their body inclination and legs movement is presented in [39]. These parameters are automatically set by a self-learning fuzzy controller that is continuously monitoring and stabilising the cardiovascular parameters of patients, such as heart rate and blood pressure, to safe ranges. The proposed system aimed to effectively rehabilitate the deconditioned cardiovascular system following a long duration of bed rest in bed-ridden patients. Although the system was tested with healthy subjects, the cardiovascular parameters were successfully controlled within medically tolerable ranges during the realisation of early mobilisation exercises. The results were promising enough to study the suitability of the system in bed-ridden patients over prolonged periods. Similarly, ref. [40] proposed *Erigo*, a novel tilt table with an integrated robotic stepping mechanism for passive mobilisations. The proposed system aimed to recover the cardio-pulmonary system of patients with spinal cord injury or traumatic brain injury with passive movements in the patients’ lower limbs. The customised verticalisation and stepping mechanism of the device enabled, for the first time, the generation of physiological load patterns. Although the results demonstrated a direct effect of the passive moment on the circulatory system, further studies with more patients and evaluating the long-term effects of the therapy are needed.

The authors in [35] proposed a pioneering wearable suit prototype aiming to provide whole-body mechanical vibrations, which are an effective treatment in rehabilitating immobilised patients to prevent muscle atrophy. Despite the benefits of whole-body vibration, there is a lack of technological solutions. This prototype was capable of delivering vibrations at multiple frequencies simultaneously to different parts of the body using inertial actuators. The results demonstrated an increase in muscle activation, tissue oxygenation and oxygen consumption. These increases were similar to those observed during mild physical activity, hence becoming a suitable solution to combat muscle weakness. However, these results were obtained from healthy patients, and its efficiency in critically ill hospitalised patients is still unknown. The authors in [38] developed a wearable mechatronic elbow trace to quantify the muscles health from electromyography (EMG) signals in musculoskeletal injured people. More specifically, six bipolar electrodes were placed over the patients’ arms, and characteristics from EMG signals during the realisation of different exercises were extracted. By comparing the results between a group of healthy people and a group of people with a musculoskeletal injury or disorder in their elbows, the authors observed statistically significant differences in the magnitudes of muscle recruitment and activation between the two groups. Moreover, authors also noticed differences in the signals of injured patients according to their therapy stage: patients at the end of their therapies show similar trends closer to those of the healthy population. Hence, the healing progress changes the muscle activation patterns, which could ultimately lead to the improvement of therapy for rehabilitation robots.

Indeed, robotic-assisted therapy is increasingly used in the EM field, from walking exoskeletons to end-effector robots, including smart mechatronic wearable systems that aim at performing passive mobilisations [29,31]. They are appealing for EM as they can facilitate repetitive and intensive tasks training, provide assistance when needed, as well as deliver feedback. However, their high cost can be a barrier to their integration into EM protocols.

#### 3.1.3. Sensors to Monitor EM Routines

Technology can also be used to improve the monitoring of EM routines. In this line, e-PEMICU is presented as an e-Health platform to support EM [37]. This solution, inspired in recent healthcare paradigms [17], is founded on the use of non-invasive motion sensors, smartphones, and further mobile devices to enhance ICUs in a cost-efficient and minimally intrusive manner. In addition to managing and recording EM sessions, practitioners can also objectively measure the degree of achievement of the performed routines as well as to properly analyse the collected data with data mining or process mining algorithms. The prototype was tested in a hospital environment, although no quantitative results were provided. In fact, the use of actigraphy is gaining the attention of professionals in the ICU and EM practitioners. According to [32], 16 studies describe the use of actimetry sensors (typically based on accelerometers) to measure patient activity in ICU settings.

Tsukamoto et al., in [41], describe the development of a quantitative monitoring device for rehabilitation training. The device, consisting of two tri-axial accelerometers attached to the patient’s head and waist, enabled estimating the locus of patients’ movement. By comparing healthy and hemiplegic patients, the authors observed differences in the height change at the head and at the waist, as well as the tremble in patients caused by the weakness of strength of the patients’ legs. The main advantage of the suggested solution is that, as the patient’s motion is depicted as graphics animations, therapists can easily understand the effectiveness of EM.

The proposal in [42] focuses on early rehabilitation of patients with acute myocardial infarction during their bed rest. Authors estimated the body movement of patients using several non-invasive temperature sensors and observed a positive correlation between an increased number of body movements with an increased heart rate. Hence, this measurement could be used to evaluate the physical activity for each recovery stage quantitatively.

Demrozi et al., in [36], studied the relationship between activity intensity and pain intensity in critically ill patients hospitalised in surgical ICUs. These measurements are essential indicators to assess the recovery progress of patients, maximise mobility and physical function, and reduce the prescription of opioids and other pain-relief medication. Unfortunately, authors detected a lack of studies examining this relation, and when conducted, measurements were manual and sporadic, limiting the extraction of actionable knowledge. On the one hand, activity intensity information was collected using three actigraphy devices, equipped with a tri-axial accelerometer sensor, placed on the wrist, arm and ankle. Furthermore, on the other hand, pain intensity was assessed hourly using the DVPRS scale (Defense and Veterans Pain Rating Scale, a scale that grades pain with a number, from 0 “No pain” to 10 “As bad as it could be, nothing else matters”), a well-known clinical practice. Although the results showed a relation between mild pain levels and high activity levels for the majority of the patients, some of them exhibited severe pain at low activity levels, indicating the need for optimising their early mobilisation routines.

Finally, the survey in [27], analyses 32 articles published in 2020 and focuses on several experiences that use telerehabilitation to deliver and monitor low-intensity EM programmes. Several proposals focused on COVID-19 patients and based on the use of tablets and motion sensors support that telerehabilitation is an important alternative to face-to-face exercises, without increasing the exposure and risk of contamination of practitioners [43,44,45,46].

## 4. Discussion

In this section, we first overview the limitations of the analysed articles. Moreover, we propose and discuss the concept of a smart healthcare-enhanced EM. Finally, we overview some existing proposals out of the scope of EM that could be integrated into our smart health-enhanced EM.

### 4.1. Limitations of the Analysed Proposals

According to our analysis, there is a reduced number of articles on EM that consider the use of electronic devices and ICT. Moreover, the existing proposals on solutions from the ICT area applied to EM merely address the use of a single technology (e.g., tilt tables, NES, etc.), but they do not present a holistic solution to improve EM practice. In this line, the proposals in the selected articles in Sets 1 and 3 describe technology-focused settings and the effects of using a specific kind of technology but, unfortunately, do not describe how the EM practise could be enhanced with techniques such as data mining, process mining or state-of-the-art machine learning. Only [37] suggests the use of artificial intelligence and the acquired data to assess the degree of achievement and adherence to the scheduled routines.

Notwithstanding, the reviewed technologies could be integrated into a global scenario for EM, enhanced via the adoption of the smart healthcare paradigm. Moreover, some of the proposals that were initially selected for Set 3 but were filtered out during the screening phase could be considered for this scenario.

### 4.2. Smart Healthcare-Enhanced Early Mobilisation

With the smart healthcare paradigm in mind, practitioners and patients could benefit from highly personalised programs, which consider patient physiological parameters as well as the context: the exercises conducted during EM programmes could be objectively monitored with a number of sensors attached to the patient’s body, whose signals (e.g., collected from accelerometers) could verify whether the exercises are performed correctly. Besides, these sensors, data can be used to evaluate the evolution of patients and their adherence to the scheduled programmes, as well as to analyse the outcomes from a global perspective. Robotic-assisted therapy could be used to apply passive mobilisations. Additionally, the use of advanced data analysis techniques, such as artificial intelligence, data mining or process mining, may detect similarities or trends among patients, whose EM programmes could be slightly adjusted with personalised recommendations using the high-level knowledge acquired. The automatic real-time feedback received from sensors data could significantly shorten recovery times and minimise the PICS side-effects.

**Figure 3:**
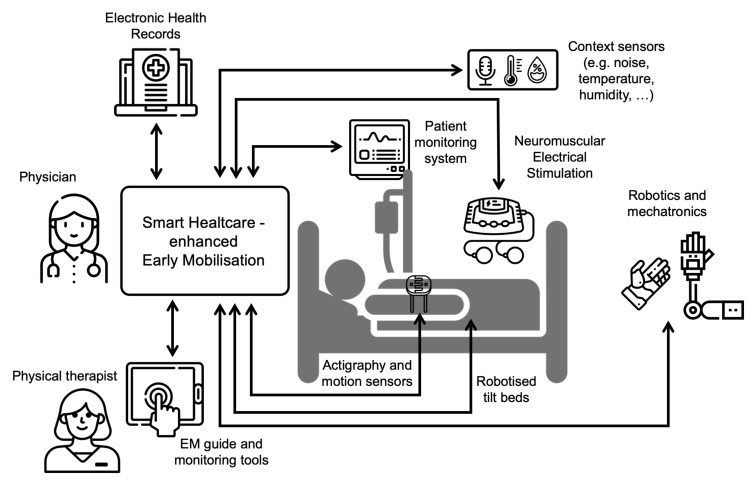
The components of a smart-healthcare-enhanced Early Mobilisation system.

Figure 3 depicts a smart-healthcare-oriented scenario for EM. In this setting, physicians count with a system that considers input data (e.g., Electronic Health Records, physiological and context parameters acquired in real-time, activity data from actigraphs and motion sensors, etc.) to personalise the activities of the EM protocols.

Unfortunately, there are several shortcomings to overcome related to the connection and interoperability of these devices with information systems. One of them is the potential variety of devices involved in such systems. In this line, ref. [47] introduces an open-source middleware framework for communication with medical devices and an application using a middleware named *Intensive Care Window*. It enables communication with intensive care unit bedside-installed medical devices over standard and proprietary communication protocols. It facilitates the acquisition of vital signs and physiological parameters from medical devices and sensors. In addition, it provides tools for annotation, visualisation and analysis. Besides interoperability, issues in the physical layer must be taken into account: in such highly sensorised indoor settings, the coexistence of multiple wireless systems requires carefully considering potential interferences, strong multipath components and signal fading [48,49]. Another shortcoming is related to information security and privacy: devices and information systems store and transmit sensitive data (e.g., health parameters along with patient identifiers might be leaked upon attacks), and hence, several countermeasures must be considered, namely the use of authentication and encryption mechanisms for information security and pseudonymisation and other privacy enhancing technologies for data privacy [50,51,52].

To improve the application and outcomes of EM programmes, the design of a smart-healthcare-enhanced ICU and, especially, the conception of the patient room must consider the well-being and experience of patients, staff and visitors [53]. The design of the room should focus on functionality, and it needs to be capable of supporting the full scope of medical informatics (from devices to information systems). In addition to the emotional welfare of patients, the success of personalised EM programmes is also greatly impacted by the room’s context. In this line, the acquisition of context data must be accompanied by a smart adaptation of the environment: sound-absorbent materials on the walls and ceiling, minimisation of sound alerts or usage of alternative vibration-based systems, natural lighting (or artificial ambient light systems to emulate day/night cycles), individual controls for temperature and humidity, etc., are essential in a successful smart healthcare scenario.

In our proposed solution, physical therapists and other users count with an electronic EM guide (in the illustration, operated using a tablet) with a schedule of mobilisations, and a monitoring tool to assess the precision and progression of the therapy. These software applications help overcome some of the provider-level barriers mentioned in Section 1.1 related to the management and communication between practitioners (i.e., team collaboration, data collection and feedback) as well as institutional-level barriers (e.g., unclear guidelines).

The EM guide could include video demonstrations of the mobilisations, so as to provide other caregivers and even patient relatives, with precise instructions on how to perform active mobilisations. As a result, a reduced group of physical therapists would be necessary to monitor and manage a growing number of personalised EM programmes without significantly having to increase resources. As a result, smart healthcare can help overcome the limited staff barrier to EM and, in the long term, promote its sustainability.

Besides physical therapists applying mobilisations by themselves, robotised elements may perform passive mobilisations to limbs, whereas NES can be applied at a specific pace and regulated according to patients’ current vital sign status and response.

### 4.3. Integrating Proposals from the Rehabilitation Area

During the phase of literature identification, we discarded proposals that, although describing experiences based on technology, did not address EM programmes and practice. However, they are worth being considered for the integration into smart-healthcare-enhanced EM scenarios.

Several of the literature articles that have not been selected are those that apply rehabilitation to patients who have suffered a stroke or patients with fractures or bone surgeries. Some of these articles present very specific rehabilitation equipment designs. For example, in [54], the design of a robot to rehabilitate after a knee operation is presented. In [55], a robotic sock (i.e., soft tissue) is used to rehabilitate the lower limbs and prevent thrombus due to lack of movement in patients who had a stroke. In [56], the use of an exoskeleton in the form of a glove for hand movements is described. The proposal in [57] combines the generation of electrical impulses to stimulate the exercises provided by an active robot on the patient’s legs. Passive mobilisation using robotics is addressed in [58]. These kinds of proposals have a great impact on rehabilitation that is focused on a specific part of the body and could be easily integrated into smart-healthcare-enhanced EM.

EM protocols may not be only circumscribed within the ICU: once the patient is discharged from the ICU or the hospital, telerehabilitation could be used to continue with exercises and activities, whereas the progression of the physical and mental state of patients, as well as their adherence to the protocols, could be monitored. In [59], a home telerehabilitation system is presented. Sensor bands are placed on patients’ arms, which capture the movements that are sent to a remote system for further analysis. Similarly, ref. [60] uses sensors to capture patients’ movement in lower limbs exercises. In this case, the movement of the patient can be visualised in an application that provides real-time feedback while performing the exercise. Remote postoperative rehabilitation of lower limbs is addressed in [61], where a new smartphone application based on plantar pressure analysis is developed and tested.

Adherence to EM protocols can be improved through gamification: it encourages patients motivation, and hence, better loyalty in treatments is achieved [62]. Game-based rehabilitation is also explored in [63], where patients have to handle a cup and achieve milestones with it while controlling the movement they make with their arm. In [64], a set of sensors detect the movement of the feet, and this translates into the interaction in a game that works on a tablet. In [65], authors describe a virtual reality application that simulates a kitchen environment: patients use a robot to perform specific actions with their upper limbs, and these movements are transferred to the virtual kitchen. In the field of pulmonary rehabilitation, accelerometers have been used for monitoring physical activity and providing feedback that may increase patient motivation [66]. Finally, in [67], authors present a robotic design that allows stimulating the patient’s gait despite being in bed and allows performing active and passive mobilisation. These articles, which focus on the patient motivation, show the technology needed to build these systems, and measuring their cost/benefit would be of interest, i.e., does the cost of building complex virtual reality systems improve rehabilitation performance accordingly?

## 5. Conclusions

The dependency on technology, ranging from sensors and electronic devices to advanced analytical techniques, is ever increasing in the healthcare industry. Care providers and healthcare organisations rely on emerging technology for improving patient care, shortening diagnosis and treatment times and decreasing healthcare expenditure in the long term. Besides, the emergence of novel healthcare paradigms based on context-awareness, such as smart healthcare, places technology as a pivotal actor in healthcare practice. Unfortunately, the role of technology in Early Mobilisation (EM) practice, an encouraging technique to shorten intensive care treatments and prevent postoperative complications in critically ill patients hospitalised in ICUs, is still in its infancy. Although the benefits of EM are well-known, the literature mainly focuses on the empirical results of EM, the impact of EM in the healthcare facilities and the main barriers to be addressed and relegate the integration of technology into EM practice to a secondary level. To fill this gap, this article has reviewed the relationship between the EM practice described in the literature and the use of technology. Moreover, with the smart healthcare paradigm in mind, a smart healthcare-enhanced early mobilisation scenario has been proposed by describing the main components and actors to be considered over the development of the solution.

More specifically, the motivation of this article revolves around three main cornerstones, namely Q1, Q2 and Q3, as described in Section 1.3. First, regarding Q1 (does the relevant literature on EM practice consider the use of technology?), we have observed that the relevant literature on EM practice does not consider the use nor the impact of technology. Indeed, recent surveys on the topic place little emphasis on technology and, when discussed, only machinery or technological applications are briefly detailed. Occasionally forgotten, neuromuscular electrical stimulation (NES)-based technology, robotics, mechatronics and sensors are the main technological solutions that can significantly contribute to enhance EM practice. This observation seems controversial with the maturity of many technologies already applied in other clinical daily practices. For instance, the use of NES within the EM practice is studied in [34], whereas the general use of technology is addressed in [26]. However, the latter does not mention the use of technology, such as sensors for monitoring the EM practise, nor robotics to help perform mobilisations.

Second, despite the above, we have identified a significant number of original research articles/studies on the application of technology to EM. Hence, concerning Q2 (does the literature on ICT include applications to EM?), we can state that there is certain interest in the engineering area to provide solutions to improve EM practice. Besides, it is worth noting that most of these works have been published in the last five years (five out of the eight reviewed articles have been published from 2015 onwards), thus demonstrating that the synergies between EM and technologies are gaining momentum. Notwithstanding, no proposal addresses integrating different technologies to contribute to the EM practice globally.

Furthermore, third, with regards to Q3 (how can technology help overcome barriers to EM practice?), we have proposed a novel solution to integrate existing solutions in the fields of engineering and ICT into a platform for smart healthcare-enhanced EM. This platform includes information sources (e.g., Electronic Health Records, real-time data from patient and context), sensorised monitoring of EM activities and solutions, such as NES and robotised elements, to help perform EM protocols. Our approach can help overcome the provider and institutional level barriers mentioned in Section 1.1, related to guidelines, management and communication between practitioners. Last but not least, our proposal can integrate solutions from the rehabilitation area, concerned with the continuity of the EM programmes in the hospital rooms or even at home, and proposals aiming at enhancing patients adherence.

All things considered, we believe that the smart healthcare paradigm can significantly contribute to improving EM and help overcome some of its barriers, especially those related to the lack of resources and management. Hence, we sustain that envisaging a smart healthcare-enhanced EM solution, working on its deployment and assessing its performance and cost/benefit would be of great interest for the medical community. Notwithstanding, the research community is only scratching the surface of the many opportunities within this field. Hence, future work will focus on the maturity of further smart-healthcare-based EM solutions from a technical perspective and addressing subsequent issues, such as interoperability, wireless channel characterisation, ethics, information security and data privacy. Latter, the implementation and validation of these solutions in real settings will be paramount to assess the benefits and suitability of technology into EM.

## Figures and Tables

**Table 1:** A summary of the results after the screening phase, with publication years and number of citations in May 2021. The results are classified according to the kind of technology they consider: neuromuscular electrical stimulation (NES), robotics and mechatronics and sensors. Papers [25,26] approach technology from a global perspective.

Set	Title	Year	Citations	NES	Robotics	Sensors
Set 1. Surveys on EM mentioning technology	Early mobilization and physical exercise in patients with COVID-19: A narrative literature review [27]	2021	0	•		•
	Rehabilitation to enable recovery from COVID-19: a rapid systematic review [28]	2021	0	•		
	Review: How Can Intelligent Robots and Smart Mechatronic Modules Facilitate Remote Assessment, Assistance, and Rehabilitation for Isolated Adults With Neuro-Musculoskeletal Conditions? [29]	2021	0		•	
	Three-Fourths of ICU Physical Therapists Report the Use of Assistive Equipment and Technology in Practice: Results of an International Survey [25]	2021	0			
	The effects of neuromuscular electrical stimulation in critically ill patients: A systematic review and meta-analysis of randomised controlled trials [30]	2020	6	•		
	Gait rehabilitation after stroke: review of the evidence of predictors, clinical outcomes and timing for interventions [31]	2020	3		•	
	Actigraphy to Measure Physical Activity in the Intensive Care Unit: A Systematic Review [32]	2019	8			•
	Physiotherapy in the neurotrauma intensive care unit: A scoping review [33]	2018	4	•		
Set 2. The most-cited contributions on EM that mention the use of technology.	An early rehabilitation intervention to enhance recovery during hospital admission for an exacerbation of chronic respiratory disease: Randomised controlled trial [34]	2014	144	•		
	Technology to enhance physical rehabilitation of critically ill patients [26]	2009	127			
Set 3. Original research articles on the application of technology to EM	The Effect of Multi-Frequency Whole-Body Vibration on Muscle Activation, Metabolic Cost and Regional Tissue Oxygenation [35]	2020	0		•	
	Joint Distribution and Transitions of Pain and Activity in Critically Ill Patients [36]	2020	0			•
	e-PEMICU: an e-Health Platform to Support Early Mobilisation in Intensive Care Units [37]	2019	1			•
	Postoperative healing patterns in elbow using electromyography: Towards the development of a wearable mechatronic elbow brace [38]	2017	1		•	
	Real-Time Closed-Loop Control of Human Heart Rate and Blood Pressure [39]	2015	11		•	
	Novel tilt table with integrated robotic stepping mechanism: design principles and clinical application [40]	2005	14		•	
	Visualization of transfer motion based on accelerometry data in the hemiplegic patients [41]	2002	0			•
	Evaluation of bed rest using a bed temperature monitor after acute myocardial infarction [42]	1995	1			•

## Data Availability

The data on the screening phase of selected works is available on http://www.smarttechresearch.com/shealthEM accessed on 6 August 2021.
